# Matrix Metalloproteinase-9 (MMP-9) Gene Polymorphism in Stroke Patients

**DOI:** 10.1007/s12017-015-8367-5

**Published:** 2015-09-02

**Authors:** Kinga Buraczynska, Jacek Kurzepa, Andrzej Ksiazek, Monika Buraczynska, Konrad Rejdak

**Affiliations:** Department of Neurology, Medical University of Lublin, Dr K. Jaczewskiego 8, 20-954 Lublin, Poland; Department of Medical Chemistry, Medical University of Lublin, Lublin, Poland

**Keywords:** MMP-9, Stroke, Type 2 diabetes, C(-1562)T polymorphism, Risk allele

## Abstract

Matrix metalloproteinases (MMPs), endopeptidases degrading extracellular matrix, play an important role in the pathogenesis of atherosclerosis and vascular disease. The aim of this study was to evaluate the association between the C(-1562)T functional polymorphism in the MMP-9 gene and risk of stroke. We examined 322 patients with stroke and 410 controls. In the patient group, 52 % had type 2 diabetes. All subjects were genotyped for the C(-1562)T polymorphism by polymerase chain reaction and restriction analysis. A significant increase in T allele and CT + TT genotype frequencies was observed in patients compared with controls (OR 1.73, 95 % CI 1.34–2.23 and 1.89, 95 % CI 1.39–2.56, respectively). The T allele carriers were younger at the onset of stroke (63.5 ± 11.7 years) than patients with CC genotype (71 ± 14.1 years) (*p* = 0.0002). The comparison between patients with T2DM and without it showed that the T allele and CT + TT genotype were more frequent in T2DM patients (OR 1.48, 95 % CI 1.03–2.12 for T allele and 1.44, 95 % CI 1.93–2.24 for CT + TT genotype). In conclusion, our findings suggest that MMP-9 C(-1562)T polymorphism is significantly associated with risk of stroke in patients with and without T2DM.

## Introduction

Matrix metalloproteinases (MMPs), well-known inflammatory mediators, belong to a family of structurally related zinc-binding proteolytic enzymes that are widely distributed in human tissues. They degrade almost all components of extracellular matrix in both physiological and pathological processes (Visse and Nagase 2003). MMPs are involved in the pathogenesis of atherosclerosis by the activation of migration and proliferation of smooth muscle cells and by induction and destabilization of atherosclerotic plaques (Galis and Khatri 2002; Katakami et al. 2010). Imbalanced MMP activity has been reported in clinical conditions involving the cardiovascular and cerebrovascular diseases (Galis and Khatri 2002; Siefert and Sarkar 2012; Chen et al. 2013).

Ischemic stroke (IS) and intracerebral hemorrhage (ICH) are both associated with activation and altered expression of MMPs, mainly MMP-2 and MMP-9. Several reports have shown that serum levels of MMP-9 increase in the acute phase of stroke and MMP-2 levels increase after several days (Romanic et al. 1998; Fatar et al. 2005). Dysregulated MMP activity leads to uncontrolled degradation of extracellular matrix and basal lamina proteins with serious effects for the blood–brain barrier integrity and neuroinflammatory consequences (Candelario-Jalil et al. 2009; Kurzepa et al. 2014).

The high levels of MMP-9 in acute ischemic stroke confirm the involvement of this metalloproteinase in the regulation of inflammation in stroke (Cojocaru et al. 2012; Demir et al. 2012).

The MMP-9 gene is located on chromosome 20q12.2–13.1. Sequence analysis revealed several single-nucleotide polymorphisms (SNPs) with some of them functionally important (Zhang et al. 1999a, b). Previous studies have demonstrated that MMP-9 activity is controlled by a functional -1562 C/T polymorphism in the promoter region of human MMP-9 gene (Zhang et al. 1999a, b; Blankenberg et al. 2003; Medley et al. 2004). Using a reporter assay technique, it was shown that the T -1562 allele has higher promoter activity in driving gene expression than the C -1562 allele (Zhang et al. 1999a, b). In human aortic samples, MMP-9 mRNA and protein expression were significantly higher in T allele carriers (Medley et al. 2004).

There are only few studies that evaluated association of MMP-9 gene polymorphism with susceptibility to stroke (Kaplan et al. 2008; Katakami et al. 2010; Szczudlik and Borratynska 2010) or functional outcome after stroke (Manso et al. 2010). The aim of this study was to evaluate the association between the C(-1562)T MMP-9 gene polymorphism and risk of stroke.

## Materials and Methods

### Subjects

The study group consisted of 322 unrelated Caucasian adult patients with either a recent stroke (patients admitted to Stroke Unit of Department of Neurology) or a history of stroke (patients admitted to Departments of Neurology and Nephrology of Medical University, Lublin, Poland). There were 156 males and 166 females. Inclusion criteria were as follows: focal neurological deficit of sudden or rapid onset lasting more than 24 h (with or without acute lesion on cranial CT or MRI scans consistent with focal deficit) and a documented history of stroke.

Stroke was classified into ischemic or hemorrhagic based on brain imaging. In the patient group, 290 patients had ischemic stroke and 32 intracerebral hemorrhage. Exclusion criteria included hereditary thrombotic disease and diagnosed brain tumor. For all individuals enrolled in the study, a medical history for the period prior to enrollment, including presence of hypertension, diabetes, coronary heart disease, atherosclerosis, kidney disease and pharmacological treatment was collected.

Hypertension was defined as either a systolic blood pressure >140 mm Hg and diastolic blood pressure >90 mm Hg or current treatment with an antihypertensive drug. In the study group, 282 patients (88 %) were hypertensive. Type 2 diabetes was diagnosed according to American Diabetes Association criteria. One or more of the following conditions were met: the classic symptoms of hyperglycemia (polyuria, polydipsia, weight loss), fasting plasma glucose ≥126 mg/dl or random plasma glucose ≥200 mg/dl, the use of insulin or oral hypoglycemic agents. The mean duration of diabetes was 12.6 years (range 6–27). It was estimated from time of the first symptoms attributable to the disease or time of first detection of glycosuria.

A total of 169 patients (52 %) had diabetes. Cardiovascular disease (CVD) was diagnosed and documented as one or the combination of several pathological states: congestive heart failure, left ventricular hypertrophy, angina pectoris, ischemic heart disease, myocardial infarction, ischemic cerebral stroke, vascular calcifications or atheromatous lesions. Each clinical manifestation of CVD was confirmed by appropriate biochemical, radiographic, echocardiographic and vascular diagnostic criteria. There was a substantial overlap between categories.

Healthy control subjects (*n* = 410) with normal ECG and no clinical signs of renal, cardiovascular and neurological disease were randomly recruited among hospital staff and blood bank donors who underwent health examination. They were asked for risk factors, existing diseases and medication intake.

Written informed consent for genotyping was obtained from all subjects in accordance with principles of the 1964 Declaration of Helsinki. The study protocol was approved by the institutional ethics committee.

### Determination of MMP-9 Genotype

Genomic DNA was extracted from peripheral blood leukocytes obtained by the standard procedure. The -1562 C/T (rs3918242) polymorphism in the MMP-9 gene was analyzed by amplification of 435-bp DNA fragment by polymerase chain reaction (PCR). The following primers were used for amplification reaction: sense primer 5′-GCCTGGCACATAGTAGGCCC-3′ and antisense primer 5′-CTTCCTAGCCAGCCGGCATC-3′. Genomic DNA (300 ng) was amplified in a final volume of 30 μl using the following conditions: initial denaturation at 95 °C for 6 min, followed by 35 cycles of 94 °C for 30 s, annealing at 60 °C for 30 s and extension at 72 °C for 1 min. A final extension step was at 72 °C for 7 min. Ten microliters of the PCR product were digested overnight at 37 °C with 5 U of Sph I restriction endonuclease (Thermo Scientific, Waltham, MA), and resulting fragments were separated on a 2 % agarose gel. The fragment sizes were 435 bp (undigested) for the C allele and 247 bp + 188 bp for the polymorphic variant (T allele). The quality of genotyping was controlled by using blind DNA duplicates for random samples. In addition, 20 samples were randomly selected for each genotype and the PCR products were sequenced in CEQ 8000 Genetic Analysis System (Beckman Coulter, England). There was a 100 % concordance between genotyping assays.

### Statistical Analysis

Statistical calculations were performed using SPSS version 11.0 for Windows (SPSS, Inc., Chicago, IL, USA). For baseline characteristics, the normally distributed continuous variables are presented as mean ± SD. The Hardy–Weinberg equilibrium was assessed using a Chi-squared test with 1 degree of freedom. Genotype distribution and allele frequencies were compared between groups using a Pearson’s Chi-squared test of independence with 2 × 2 contingency and *z* statistics. ANOVA was used to compare average values of biochemical parameters. Pearson’s Chi-squared test and Mann–Whitney test were used for comparing discrete and continuous variables. For significant allelic and genotyping associations, the adjusted odds ratios (ORs) with corresponding 95 % confidence intervals (CI) were calculated. Since the number of the homozygous patients TT was small, we combined the TT and CT into a single group to improve the statistical power. Power calculations were performed with the program of Purcell et al. (Purcell et al. 2003) (available at http://pngu.mgh.harvard.edu/~purcell/gpc/). In the stroke patient group, the frequency of the MMP-9 T allele was 0.25. The study had 91.3 % power (*α* = 0.05) to detect an association (OR vs controls 1.73, 95 % CI 1.34–2.23). An interaction of the polymorphism with various risk factors was examined with multiple logistic regression analysis. The Bonferroni correction was applied for multiple comparisons with control type 1 error. Statistical significance was set at *p* < 0.05.

## Results

The -1562 C/T (rs3918242) polymorphism in the MMP-9 gene was genotyped in 322 patients with stroke (90 % had ischemic stroke) and 410 healthy control subjects. The demographic and clinical characteristics of all studied subjects are summarized in Table 1. The stroke patients presented an average age 10 years older than controls. Gender did not statistically differ between patients and controls (*p* = 0.31). Compared with controls, the stroke patient group had a higher prevalence of conventional risk factors for stroke, including higher levels of total cholesterol and triglycerides (*p* < 0.01 and *p* < 0.001, respectively). BMI in stroke patient group was also higher than in controls (*p* < 0.001).

**Table 1:** Demographic and clinical profile of studied subjects

Variable	Stroke patients (*n* = 322)	Controls (*n* = 410)	*p* value^a^
Male/female	156/166	217/193	0.31
Age at study (years)	67.3 ± 16.2	56.8 ± 19.2	0.012
Age at stroke (years)	64.3 ± 12.7	NA	
Diabetes mellitus (%)	169 (52)	0	
Cardiac disease (%)	219 (68)	0	
Hypertension (%)	282 (88)	0	
Atrial fibrillation (%)	90 (28)	0	
Total cholesterol (mmol/l)	4.9 ± 1.3	4.2 ± 1.7	<0.01
HDL cholesterol (mmol/l)	1.2 ± 0.6	ND	
LDL cholesterol (mmol/l)	2.91 ± 1.02	ND	
Triglycerides (mmol/l)	1.9 ± 1.3	1.21 ± 0.86	<0.001
BMI (kg/m^2^)	27.1 ± 4.2	25.8 ± 4.6	<0.001

Values are presented as mean ± SD (continuous characteristics) or as numbers with percent in parentheses (discrete characteristics)

*NA* not applicable, *ND* not determined

^a^Pearson’s Chi-squared test for categorical variables and Mann–Whitney test for continuous variables

The prevalence of MMP-9 -1562 genotype and allele frequencies in stroke patients and controls is presented in Table 2. The genotype distribution among the controls and stroke patients was in Hardy–Weinberg equilibrium (*p* = 0.536 and *p* = 0.359, respectively). A significant increase in the T allele and CT + TT genotype frequencies was observed in stroke patients compared with healthy controls (OR 1.73, 95 % CI 1.34–2.23 and 1.89, 95 % CI 1.39–2.56, respectively). Multivariate logistic regression analysis was used to assess the role of the rs3918242 genotype and other coexisting factors in stroke. These adjustments did not substantially affect the OR estimates. After confounding effects of age, gender, BMI and lipid profile were adjusted, the T allele and TT genotype were significantly associated with stroke in our study population.

**Table 2:** Genotype and allele distribution of C(-1562)T gene polymorphism in MMP-9 gene in patients and controls

*N*	Genotypes				Alleles		OR (95 % CI)	
	CC	CT	TT	CT + TT	C	T	CT + TT genotype	T allele
Stroke patients								
322	174 (54)	132 (41)	16 (5)	148 (46)	0.75	0.25	1.89 (1.39–2.56) *p* < 0.0001	1.73 (1.34–2.23) *p* < 0.0001
Controls								
410	283 (69)	119 (29)	8 (2)	127 (31)	0.84	0.16	Ref.	Ref.

Genotype distributions are shown as numbers (%). HWE test for controls *X* ^2^ = 1.25, *p* = 0.536; for stroke patients *X* ^2^ = 2.05, *p* = 0.359

Mean age at the onset of stroke was compared in patients with CT + TT versus CC genotype. The T allele carriers (CT + TT) were younger at the onset of stroke (63.5 ± 11.7 years) than patients with CC genotype (71 ± 14.1 years) (*z* = 3.7167, *p* = 0.0002).

The patients were divided into subgroups with and without T2DM (*n* = 169 and *n* = 153, respectively). The comparison between these subgroups showed that the T allele and CT + TT genotype were more frequent in patients with T2DM compared with those without diabetes, with the OR 1.48 (95 % CI 1.03–2.12) for T allele and 1.44 (95 % CI 1.93–2.24) for CT + TT genotype (Table 3).

**Table 3:** Genotype and allele distribution of C(-1562)T gene polymorphism in MMP-9 gene in stroke patients with and without T2DM

*N*	Genotypes				Alleles		OR (95 % CI)	
	CC	CT	TT	CT + TT	C	T	CT + TT genotype	T allele
STR + T2DM								
169	84 (50)	72 (43)	13 (7)	85 (50)	0.71	0.29	1.44 (1.93–2.24) *p* = 0.101	1.48 (1.03–2.12) *p* = 0.031
STR no T2DM								
153	90 (59)	60 (39)	3 (2)	63 (41)	0.78	0.22	Ref.	Ref.
Controls								
410	283 (69)	119 (29)	8 (2)	127 (31)	0.84	0.16		

Genotype distribution is shown as numbers (%). For STR + T2DM subgroup compared with control group, OR is 2.07 (95 % CI 1.53–2.79) for T allele and 2.25 (95 % CI 1.56–3.25) for CT + TT genotype

*STR* stroke, *T2DM* type 2 diabetes

## Discussion

In the present study, we identified an association between genetic polymorphism in matrix metalloproteinase-9 gene and the risk of ischemic stroke. The main finding is that the T allele of the -1562 C/T polymorphism might increase risk of stroke. Compared with control group, the increase was 1.9-fold for stroke patients carrying T allele and 2.3-fold for diabetic T allele carriers.

The similar result was obtained by Nie et al. (2014) in a recently published study of 396 Chinese patients with ischemic stroke. The TT genotype and T allele frequencies of MMP-9 C/T polymorphism were significantly increased in stroke patients. The stroke incidence was increased 1.5-fold in patients carrying the T allele. Our results are not consistent with a previous study performed in a Polish population that showed no association between the MMP-9 -1562 C/T polymorphism and increased risk of ischemic stroke (Szczudlik and Borratynska 2010). The authors studied 418 patients with ischemic stroke of various etiologies. Statistical analysis did not show a significant difference in distribution of CC, CT and TT genotypes or C and T alleles between ischemic stroke patients and controls. The association of the MMP-9 -1562 C/T polymorphism with cardiovascular disease was found in another Polish study of 110 patients with coronary atherosclerosis (Goracy et al. 2003). The results suggested that C(-1562)T MMP-9 transition is associated with premature ischemic heart disease in Polish patients. It is difficult to explain the discrepancy between our results and those of Szczudlik et al. (Szczudlik and Borratynska 2010) since both studies involved similar number of stroke patients from Polish population. Stroke is a complex disease in which it might be difficult to demonstrate an impact of a single polymorphism. An interactive effect of several factors may lead to underestimation or overestimation of a role of given polymorphism in determining the phenotype. There are some limitations in this type of study that might account for differences in obtained results. Control subjects are considered free of cerebrovascular disease by medical history, lack of neurological deficits and laboratory examinations. However, without brain imaging studies, some control subjects might have been affected by silent stroke. This could reduce the statistical power. Another limitation is that some interactions between MMP-9 and other candidate genes might be different between different studies, but these interactions are usually not taken under consideration.

The -1562 C/T polymorphism in MMP-9 gene was analyzed in a study of four SNPs in patients with myocardial infarction (854 patients) and those with ischemic stroke (367 patients) (Kaplan et al. 2008). All studied MMP-9 SNPs or haplotypes were not associated with myocardial infarction or ischemic stroke.

Diabetes mellitus is a well-recognized risk factor for coronary and cerebrovascular diseases (Banerjee 2012; Djelilovic-Vranic et al. 2013). They share many characteristics, due to the fact that diabetes affects blood vessels and stroke is a disease of blood vessels. Moreover, diabetes is commonly associated with other cardiovascular risk factors, such as hyperlipidemia and increased low-density lipoprotein levels (Taylor et al. 2013). Therefore, MMP-9 might be one of the potential candidate genes for the link between T2DM and ischemic stroke. The allele frequencies of different polymorphisms in diabetic patients might be different from those in nondiabetic subjects. Also, different polymorphisms could be related to different risk factors in patients with type 2 diabetes. In our study, the T allele and CT + TT genotype were more frequent in patients with T2DM compared with those without diabetes, with the OR 1.48 (95 % CI 1.03–2.12) for T allele and 1.44 (95 % CI 1.93–2.24) for CT + TT genotype. This indicates that there might be a different mechanism for the effect of -1562 polymorphism in diabetic patients. This effect might depend on the population studied. Katakami et al. (2010) evaluated the -1562 MMP-9 polymorphism in 3094 Japanese type 2 diabetes subjects. They did not observe a statistically significant association between this polymorphism and the prevalence of cerebral infarction.

The mechanism of the observed effect of studied polymorphism on susceptibility to stroke is unknown. The -1562 C/T polymorphism is located in the promoter region of MMP-9 gene. Transcriptional activity of the T allele was found to be significantly higher than that of the C allele (Blankenberg et al. 2003). Increased expression of MMP-9 may increase plaque instability. The disruption of vulnerable atherosclerotic plaque and subsequent thrombus formation play critical role in the pathogenesis of cerebrovascular disease.

In conclusion, our findings suggest that MMP-9 -1562 genotypes are significantly associated with the risk of ischemic stroke in patients with and without T2DM. These results require replication in future studies. The effect on observed genetic susceptibility is modest but might be important in the presence of other genetic factors. The study provides new clinically relevant information regarding genetic determinants of susceptibility to stroke.
